# An open annotation ontology for science on web 3.0

**DOI:** 10.1186/2041-1480-2-S2-S4

**Published:** 2011-05-17

**Authors:** Paolo Ciccarese, Marco Ocana, Leyla Jael Garcia Castro, Sudeshna Das, Tim Clark

**Affiliations:** 1 Harvard Medical School and Massachusetts General Hospital, Boston MA, USA; 2 Balboa Systems, Newton MA, USA; 3 Universität der Bundeswehr München, Neubiberg, Germany; 4 University of Manchester, School of Computer Science, Manchester UK

## Abstract

**Background:**

There is currently a gap between the rich and expressive collection of published biomedical ontologies, and the natural language expression of biomedical papers consumed on a daily basis by scientific researchers. The purpose of this paper is to provide an open, shareable structure for dynamic integration of biomedical domain ontologies with the scientific document, in the form of an Annotation Ontology (AO), thus closing this gap and enabling application of formal biomedical ontologies directly to the literature as it emerges.

**Methods:**

Initial requirements for AO were elicited by analysis of integration needs between biomedical web communities, and of needs for representing and integrating results of biomedical text mining. Analysis of strengths and weaknesses of previous efforts in this area was also performed. A series of increasingly refined annotation tools were then developed along with a metadata model in OWL, and deployed for feedback and additional requirements the ontology to users at a major pharmaceutical company and a major academic center. Further requirements and critiques of the model were also elicited through discussions with many colleagues and incorporated into the work.

**Results:**

This paper presents Annotation Ontology (AO), an open ontology in OWL-DL for annotating scientific documents on the web. AO supports both human and algorithmic content annotation. It enables “stand-off” or independent metadata anchored to specific positions in a web document by any one of several methods. In AO, the document may be annotated but is not required to be under update control of the annotator. AO contains a provenance model to support versioning, and a set model for specifying groups and containers of annotation. AO is freely available under open source license at http://purl.org/ao/, and extensive documentation including screencasts is available on AO’s Google Code page: http://code.google.com/p/annotation-ontology/ .

**Conclusions:**

The Annotation Ontology meets critical requirements for an open, freely shareable model in OWL, of annotation metadata created against scientific documents on the Web. We believe AO can become a very useful common model for annotation metadata on Web documents, and will enable biomedical domain ontologies to be used quite widely to annotate the scientific literature. Potential collaborators and those with new relevant use cases are invited to contact the authors.

## Background

### Bridging the gap between ontologies and documents

Much current work in biomedical ontologies now focuses on detailed formal classification of objects, functions and processes, using description logics [1-14]. This approach creates a set of fixed categories for searching, navigating and integrating ontology-annotated content on the web, whether in the standard journal publications or in web “collaboratories” [15] or other web publication vehicles.

However, we currently lack a robust common set of methods for linking text in new biomedical publications to formal ontological elements, with full annotation provenance. This is a curious omission, as scientific publications have been the core units of work, credit, and collaboration in the scientific enterprise since approximately the end of the seventeenth century [16]. Science as a system of inquiry has functioned and evolved since that time based on the use of scientific papers as its fundamental boundary objects [17] or “literary technology” [18] and therefore scientific publication as its fundamental social activity. The purpose of this paper is to provide an open, shareable representational structure for dynamic integration of biomedical domain ontologies with the scientific document.

Basic requirements for such a structure are that it must

(a) function without update control of the annotated resource;

(b) support provenance, versioning and access control information;

(c) enable association of terms (classes or instances) in ontologies, terminologies, vocabularies and/or folksonomies, with robustly defined fragments of the annotated resource;

(d) be orthogonal to all domain ontologies; and

(e) be implemented in a form compatible with the Semantic Web.

Seminal lines of research in distributed link services (DLS) [19] and in conceptual open hypermedia (such as COHSE) [20] have explored this area, as did the Annotea project, without yet to our knowledge publishing an annotation metadata specification fully meeting requirements for annotation of biomedical research on the semantic web. In the case of COHSE, this may well have been due to an issue of timing. At the time COHSE was developed, there were far fewer semantic web resources capable of being mapped to documents that there were today, and the available technologies were relatively immature [21]. In the case of Annotea, timing was probably a factor as well. However, today we are in a situation that allows us to proceed differently.

The authors believe that a remedy for this gap falling between the available suite of biomedical ontologies and the world of scientific documents, will be of great value to the research community.

The Annotation Ontology (AO), proposed here, meets all the requirements listed above. It is orthogonal to any specific domain ontology but capable of recording associations between elements (Representational Units [22]) of such domain ontologies, and online resources such as scientific papers, images, etc. So long as the element to be associated is represented as a URI, AO is satisfied. Associations of URIs may be made to specific fragments of each resource, or to the resource as a whole, and are associated with specific annotation provenance, versioning and access information. This is stand-off annotation and does not require update control of the annotated resource.

The Annotation Ontology provides a common model for document metadata derived from either (or both) text mining and manual annotation of scientific papers. Using this ontology, such metadata can now be published as open linked data on the Web, and shared amongst interested parties, or all parties. This means the elements of domain ontologies can now link new scientific content arising as documents, across scientific specializations and their associated web communities of discourse, as the annotation is applied to specific sets of documents.

Doing this in practice requires a framework for generating document annotations with algorithmic assistance, under human supervision. We have built such a framework, which will be described in a separate forthcoming paper. The process of defining the elements and structure of AO proceeded in tandem with developing the framework and soliciting feedback from its users. The framework we have developed need not be the sole way of annotating web documents using the annotation ontology, and we hope additional such tools are built wherever needed.

We believe these tools will allow an expansion in the application of biomedical domain ontologies to the place most scientists focus: new publications and the results they report. And they are also needed to integrate findings across the emerging publication model of biomedical web communities, or collaboratories.

As researchers in, and cooperating developers of, such collaboratories [23-25], we note that – particularly in complex phenomena such as neurodegenerative disorders – there is both a requirement to focus on a single disorder, such as Parkinson’s Disease (PD) – and a requirement to be aware of relevant information from other related fields. These requirements would seem to work against each other. Why would a PD researcher read the literature on addiction? Yet both phenomena deal with dopaminergic pathways, and investigators have reported surprising crossover phenomena, such as the development of pathological addictions or impulse control disorders in PD patients overmedicated with dopamine agonists [26,27]. If the literature in both PD and addiction were automatically marked up with semantic metadata, these connections might emerge much more freely.

Content in web collaboratories has the advantage of providing a strong focus to the collected, evolving discourse. Specialists accessing material in such a focused web community – such as PD Online [24] (http://pdonlineresearch.org), or Alzforum [28,29] (http://www.alzforum.org) – will not need to wade through extraneous material on cardiology, drug addiction, hematology, and so forth. Essentially what these communities do is dramatically improve the signal-to-noise ratio for specialists, making the information explosion in science nearly tractable within a given specialty.

Annotation – either marking up contributions with comments, or more importantly, with relevant concepts and entities from biomedical ontologies – provides a technological boost to ”strategic reading“ for members of such communities [30] and selectively breaches established specialist focus boundaries and semantic barriers where required [23]. This of course also applies to subscribers to specialist journals as well.

AO’s features are specifically designed to support content linking in collaboratories, and can also be used for many other applications requiring persistent mapping of ontology elements to scientific web resources or resource fragments.

Existing ontologies and vocabularies which can serve as a basis for such annotation are particularly abundant in the biomedical field and are often expressed in OWL/RDF ontologies [31,32] or as instances in SKOS taxonomies [32]. Subjects for ontological structuring include biological processes, molecular functions, anatomical and cellular structures, tissue and cell types, chemical compounds, genes and proteins.

We take proteins as a typical example. There are a number of database resources that catalog and identify proteins. UniProt is certainly the most popular but, at the moment, is not available in OWL format (i.e. as a description logic). The PRO Ontology [2] is a project which represents a growing proportion of the content of UniProt and other protein databases as declarations in OWL, and is interoperable with other OBO Foundry ontologies - such as the Sequence Ontology [33] and the Gene Ontology [10,34] - that provide representations of protein qualities. This interoperability facilitates cross-species comparisons, pathway analysis, disease modeling, and the generation of new hypotheses through data integration and machine reasoning.

### Social tagging

AO also supports social tagging, which has emerged with the increasing use of social media in science publishing, as a kind of “bottom-up” informal terminology system completely opposite in spirit to the formal ontology, but with a similar purpose. Social tagging systems, also known as folksonomies, have become increasingly popular with the advent of Web 2.0 [35]. The process of tagging allows association of free text to web resources, thus improving their classification and organization. The simplicity and immediate benefits for end users are some of the reasons behind the acceptance of tags [35,36]. Tags improve retrieval [37], and promote social interaction by enabling the construction of social networks based on the common interests that they represent [37,38].

However, tags have certain limits: i) free text tags can have several variations, e.g morphological and spelling variations, ii) tags can be ambiguous and iii) they are usually defined in different levels of granularity according to the user expertise [39-41]. Also, folksonomies do not share a common representation; thus sharing and reusing tags can be difficult [38].

Nonetheless meta-models [42] and ontologies [43-45] for representation of social tagging exist and there are various ways of using these approaches to migrate informal tags into more formal systems. They are very important to capture as use cases because of the widespread use of social tagging and the need to integrate tagging approaches and structured terminology approaches dynamically.

### Formal semantic models and annotation of web resources

Currently, several well-controlled formal semantic models are available for use by biomedical communities. A prominent example is the OBO Foundry suite of ontologies [4], which includes the Gene Ontology [46] and other important resources previously mentioned. These can be used out of the box to semantically annotate content - providing there is a mechanism for attaching the semantic tags to the target.

In associating terms either from formal ontologies, or ad-hoc tags, with content in a scientific document, ideally it should be possible to refer to either a whole document, or to a single part of it. We take a broad view of what constitutes a document, extending beyond the normal parameters of the scientific paper, and, we hope, reflecting the nature of the emergent web-based biomedical communications ecosystem. So, we include database entries and biomedical images on the web in our view of documents. Some examples of document fragments that should be capable of independent referencing would be: a chunk of text in an html page or a section of an image, a portion of audio/video file, an entry in a database, or a portion of a table. The mechanisms to refer to document fragments have to cover multiple use cases, from the simple ones represented by very stable documents to the tricky ones where documents are constantly changing.

Electronic documents on the web are not always stable. We like them because they are “dynamic”, but this feature is also a bug. Blogs change very often, dynamic data sets produce evolving tables, materials and methods in research papers may be appended. Therefore it is important to be able to anchor the annotations so that the anchor remains stable irrespective of the evolution of the document. The Annotea Project [47,48] attempted to address this issue and allowed the definition of a context to specify the portion of the document where the tag is attached. In the same vein, Garcia et al. [49] propose a Living Document, where annotations can also be attached to an atomic component within a document and related to semantic entities as well as online resources.

Finally, it should be possible to adopt the same mechanisms for other kinds of annotation, in addition tosemantic and scoial tagging. Comments and notes are a good example. It should be possible as well to refer to a part of a document specifying, for instance, an erratum, a definition, an example related to it, or even discourse elements such as claims and hypothesis that are represented in that part of the document.

## Methods

We developed the Annotation Ontology (AO) [50][51] (http://purl.org/ao/) to meet the requirements previously stated. These and other requirements are the result of an investigation phase carried out mainly in the context of the online scientific communities developed by our group and its collaborators in the last few years [23-25,52] and of ongoing collaborations with colleagues in multiple institutions. These include (a) a major U.S.-based pharmaceutical company, (b) the NIH-funded Neuroscience Information Framework project [53,54]; and recently also (c) a major scientific publisher, as well as (d) early-stage collaborations with academic biomedical textmining groups.

Given these requirements, and our approach consistent with semantic web principles to re-use and extend previous work wherever possible, we determined the best starting point in the Semantic Web world to be the work done in the Annotea Project [47], a seminal research project of the World Wide Web Consortium (W3C, http://w3.org) dating from the early 2000’s. The Annotation Ontology presented here was strongly influenced by an analysis of strengths and weaknesses of the Annotea work.

Also, consistent with the social tagging use case previously discussed, we determined to provide integration with existing semi-formal ontologies such as SIOC (Semantically-Interlinked Online Communities) [55], Newmann’s Tagging Ontology [44], and MOAT [56].

We also decided to support the attachment of comments and notes, and of course the annotation of hypotheses, claims, and evidence, as elements of the SWAN discourse ontology [52]. This approach offers a range of annotation possibilities, from very dynamic but with less structure and information content, to less dynamic but with more information content and structure.

**Figure 1:**
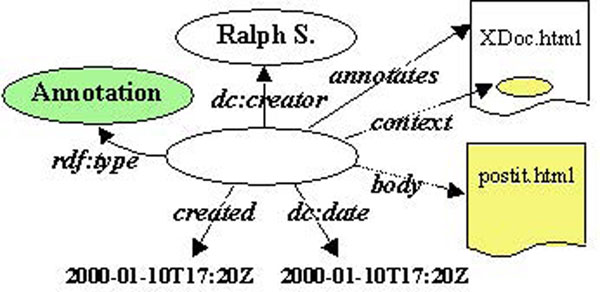
The Annotea RDF model for an annotation.

Annotea was developed as a Web-based shared annotation system based on a general-purpose open RDF infrastructure [57] where annotations are modeled as a class of metadata. Annotations, identified by a URI, are viewed as statements or remarks made by an author about a Web document. In the Annotea model (see Figure 1), the context defines where exactly inside the document the annotation is attached, and the body is a link to the content of the annotation identified by a URI and conceived to contain textual or graphical content.

Annotations are external to the documents and can be stored on one or more annotation servers responsible for controlling access – this allows us to implement local/private and remote/shared annotations. Annotations in Annotea are typed and users can classify them while creating them (examples: comment, example, explanation, change,...).

The Annotea project re-uses existing W3C technology including RDF, Xpointer [58], Xlink [59], and HTTP [60]. The XPointer mechanism is used to identify the context within a document and works well for unchanging documents but with documents that go through revision, it is possible to end up with orphan annotations or annotations that are pointing to wrong places.

**Figure 2:**
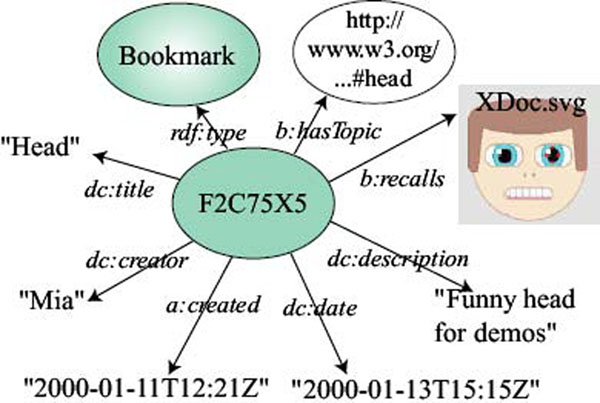
Annotea Bookmark model.

Besides the Annotation, the concept of a Bookmark was also defined by the Annotea project [61]. Annotea assumed the body of the Annotation to be an XHTML document. Bookmarks are metadata about Web documents or other resources and they are associated through the relationship hasTopic to an instance of the class named Topic defined as: an informal category for the purpose of classifying bookmarks. Topics may have subtopics and may sometimes also refer to categories in more formal ontologies. Bookmark shares a similar structure with Annotation (see Figure 2).

## Results

### The annotation ontology: core features

The core of AO is inspired by the Annotea model with a few important differences:

■ In AO we do not distinguish between Annotation and Bookmarks as we find the distinction confusing. The two concepts are combined in one single generalized model defined as Annotation Core detailed in the following sections of this paper.

■ In AO the goal is to be able to refer to several kinds of documents and document parts. Therefore, the context is defined through a more complex mechanism making use of instances of subclasses of the class Selector.

■ In AO it is possible to create annotation types in two different ways: by sub-classing as done in Annotea and by composition. The latter mechanism is introduced in AO for improving integration with already existing ontologies.

Several other features have been conceived to extend the original Annotea (Annotation and Bookmark) model in particular for allowing annotation curation – crucial for our scientific communities - and for improving annotation management and publishing.

One of the original requirements for AO was to be able to annotate content computationally with editor supervision. This requirement was identified in the early stages of the project while developing AlzSWAN (http://hypothesis.alzforum.org/swan/) – the SWAN platform for Alzheimer’s Disease developed in collaboration with AlzForum (http://www.alzforum.org/) and online since August 2008 - and StemBook (http://www.stembook.org/), online since September 2008. We later determined that multiple web communities needed to share their annotation metadata, which required a formal specification and a second-generation annotation tool.

Furthermore, we desired to incorporate a key advance over the first-generation AlzSWAN authoring tool: to reference discrete sections of any web document positionally with the annotations. Lastly, we incorporated annotation sets so that the same annotated documents could target multiple use cases. To support these features we need to go beyond Annotea. The formal metadata specification we developed allows us to define and localize document associated ontology terms and several other kinds of annotation such as comments, notes, examples, errata and definitions. The annotation is stored in a mySQL database (for performance reasons), which mirrors a set of triples instantiated according to AO. Triples and database are stored separately from the documents.

### The annotation ontology: modules

The Annotation Ontology has a modular architecture with the following list of modules:

■ Core: represents the heart of AO and collects the core elements such as Annotation, the abstract Selector, AnnotationSet and AnnotationDocument.

■ Selectors: lists the selectors we provide for identifying document fragments. The list of selectors can be easily extended according to the specific needs.

■ Types: lists all the annotation types we provide. The list of annotation types can be easily extended following simple extension rules.

■ Integration with Annotea: the triples connecting the two are collected in a separate module/file to keep the core of AO independent.

■ Integration with FOAF: all the dependencies or connections between the FOAF vocabulary and AO have been collected in this module. This choice has been motivated by the fact that not all our collaborators use FOAF for the representation of agents and documents. Therefore, we wanted to guarantee independence of AO from FOAF.

Table 1 shows the namespace prefixes used in subsequent sections of this paper both in pictures and in text. Classes and relationships are represented with font Italic.

**Table 1:** Namespace prefixes.

Core	ao	http://purl.org/ao/
Selectors	aos	http://purl.org/ao/selectors/
Types	aot	http://purl.org/ao/types/
Integration with Annotea	aoa	http://purl.org/ao/annotea/
Integration with FOAF	aof	http://purl.org/ao/foaf/
Provenance, Authoring and Versioning	pav	http://purl.org/pav/2.0/
Swan Agents	swan-agent	http://purl.org/swan/2.0/agents/
Swan	swan	http://purl.org/swan/2.0/
PRotein Ontology	PRO	http://purl.org/obo/owl/PRO#
Annotea Ontology	ann	http://www.w3.org/2000/10/annotation-ns#

When classes and relationships do not have a prefix they are intended as part of the AO core module and therefore implicitly have the ‘ao’ prefix.

In the Discussion section we demonstrate how AO is used for annotating an entire document to provide an overview of the ontology. In the following sections we will describe the different modules in detail.

## Discussion

### Annotation of an entire document

**Figure 3:**
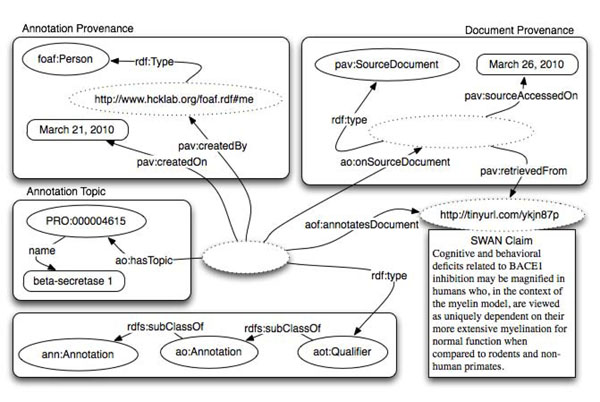
Example of annotation of an entire document with Annotation Ontology (AO).

The simplest example of annotation is depicted in Figure 3, where an entity from the PRotein Ontology (PRO) [2] has been linked. The predicate *ao:hasTopic* has been used for this purpose. By design, *ao:hasTopic* allows either classes and individuals as its range. This is to support the use of both "classes-only" ontologies such as PRO of the OBO Foundry, and/or SKOS-like terminologies whose terms are instances, as annotation. We are aware that this approach could lead to an OWL Full representation but we decided not to transform the predicate into an Annotation Property. In fact, we don’t want to preclude, for those who are interested, OWL DL reasoning and querying. Instead, we recommend our users, in case of reference to classes, to express the relationship as a restriction and therefore a valid DL statement.

The dashed ovals in the diagram represent instances while the remaining ovals represent classes. In this case annotation – an AlzSWAN claim http://tinyurl.com/ykjn87p concerning the protein ‘Beta-Secretase 1’ – is linked to a whole document. In this particular case the annotation is of type *aot:Qualifier* that states the document is related to the linked entity. Annotation types will be extensively discussed in a subsequent section. It is important to notice that it is trivial to apply the same annotation to multiple documents simply linking more documents to the annotation instance.

### Annotation provenance

AO uses FOAF [62] as the preferred way for representing agents and documents. However the integration between the two ontologies is performed through a module that is external to the core of the annotation ontology. This guarantees the independence of AO from FOAF allowing alternatives to users of AO. When FOAF is used, as in the rest of this paper, the AO annotation can be created by an agent (*foaf:Agent*): a person (*foaf:Person*), a software agent (*swan-agent:Software*), a group (*foaf:Group*) or an organization (*foaf:Organization*) annotating a resource. Annotation provenance is represented using the PAV (Provenance Authoring and Versioning, http://code.google.com/p/pav-ontology/) module of the SWAN ontology [52,63]. Resources that are the subject of an annotation are mainly documents (*foaf:Document*) but they can be any online resource. Alternatively some provenance can be provided through Dublin Core or Dublin Core Terms (http://purl.org/dc/elements/1.1/). We recommend using them not as an alternative but as add-on - PAV remains the first choice for AO due to its wide offer of features for curation that is particularly important for scientific communities. Curation will be discussed later in this paper.

### Source document provenance

In Figure 3 we see that the Document (*foaf:Document*) is linked twice to the annotation - once directly through the Annotea relationship *aof:annotatesDocument* and once through a *pav:SourceDocument* class defined in the version 2.0 of the PAV ontology [64]. FOAF defines – or better loosely defines - a *foaf:Document* class as: *The Document class represents those things*, *which are*, *broadly conceived*, *'documents'.* In AO, user needs to point to the document that he is annotating and typically this happens by means of a URI. Unfortunately, often the document changes over time but the URI stays the same. Using a property such as *pav:accessedOn* is useless if the URI is always the same as we will end up with a URI and multiple access dates with no way to determine the date is associated with each annotation. The solution we propose, which at first glance seems to introduce some redundancy, is to have a stable URI for the webpage and a different URI for each detected version of the document. To keep compatibility with FOAF, Figure 3 would translate into the following RDF:

Through this approach it is now possible, for an annotation to point both to the URI of the webpage (*foaf:Document*) and to the URI of the specific version of the document (*pav:SourceDocument*).

### Annotation core and selectors

In this section, we describe how the core and selectors of AO are used to attach annotations to various types of documents. In the context of online scientific communities, resources targeted by an annotation can be HTML pages, documents, images, videos, databases and fragment or sections of them. This variety of possible targets – not all of them with a definition of how to construct a fragment URI - and their mutability motivates the introduction of the class *Selector*.

A *Selector* identifies a portion of a resource, and may work differently for different types of documents and content types. Selectors are meant to be stable URIs. It is also common to provide different selector models for the same resource type. For instance, for selecting a chunk of text in a XHTML document we can use mechanisms based on XPointer, or one based on an offset and range, or other more robust mechanisms, like a combination of them. In fact, for immutable content selectors of the type XPointer or offset and range might be easier to deal with. In general, though, it is well known that not all HTML pages are immutable and some sections of these pages may vary with time – news and advertisements are often embedded in the document - requiring more reliable and customized fragment identification mechanisms. In AO we use the Annotea property *ann:context* to attach an XPointer to an annotation instance as shown below:

In AO, we also provide a *aos:XPointerSelector* that allows the use of XPointers and has additional benefits of a *Selector*, including the ability of defining the document provenance through an instance of *pav:SourceDocument*.

**Figure 4:**
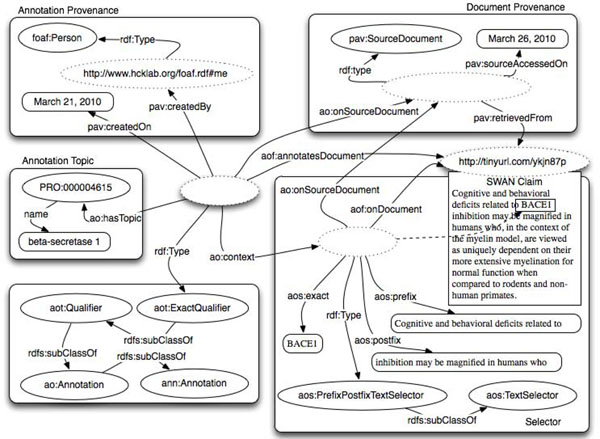
Annotating a document fragment identified by PrefixPostfixTextSelector via the properties prefix, exact match and postfix.

Figure 4 provides a similar example to Figure 3, but instead of annotating the whole document, only a portion of it has been annotated. The fragment is detected through a type of *aos:TextSelector* that identifies the exact piece of text specifying its prefix and its postfix. Also, while the annotation in Figure 3 was created manually by a *foaf:Person*, the annotation here depicted has been created by a text mining service. The annotation type is *aot:ExactQualifier* as the chunk of text is representing the protein beta-secretase 1.

Selector has several sub-classes that can be chosen based on the nature of the source document (images, videos, records in databases and text). Figure 4 illustrates an example of annotating a portion of a text document - *aos:PrefixPostfixSelector*, a subclass of *aos:TextSelector* – a subclass of *Selector*. Such selector works through three properties: the *aos:exact* is equal to the exact string or sequence of characters being annotated. The *aos:prefix* and *aos:postfix* are defined as the sequence of characters preceding and following the match. The three properties are defined ignoring any HTML/XML markup and normalizing the white spaces. The concatenation of the values of *aos:prefix*, *aos:exact* and *aos:postfix* is used to identify the section of text.

The above selector allows us to identify a portion of the document text, and link it with a term from a formally defined vocabulary. This specific selector works particularly well when the document is not immutable. In fact, even if other sections of the document change, it is possible to still detect the context if the annotated content is still present.

The RDF generated is shown below:

Several selectors can be defined to cover different use cases.

**Figure 5:**
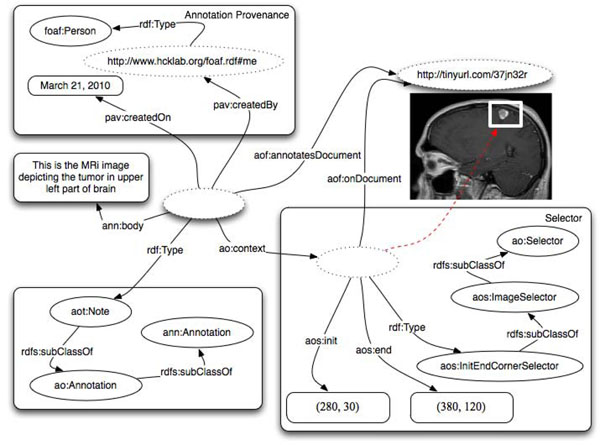
Annotation on part of an image using aos:InitEndCornerSelector to specify rectangular section in pixel units.

Figure 5 depicts an example annotation where a rectangular section of an MRI image depicting a brain tumor is selected. The selector identifies the portion of the image through specifying the coordinates of a rectangular box. In this particular case the reference to the specific version of the document through pav:SourceDocument has been omitted. This has been done to simplify the picture and it would normally be acceptable only if the image were immutable.

AO users have the flexibility of extending the selector class based on their particular use cases (instruction available on the wiki page). To allow the community around AO to grow coherently and enable interoperability, we recommend contributing the new selectors back to the AO project.

It is important to notice that multiple instances of the class *Selector* can be attached to each annotation item. This allows performing annotation of multiple targets located in the same document. The same mechanism allows the annotation of multiple targets located in different documents.

### Annotation types

In Annotea, users can create additional sub-types of annotations by using sub-classes of the Annotation class. Through this mechanism, it is possible, for instance, to introduce ‘note’ where the purpose is not to attach a term but to attach an explanatory text to a portion of a document. The list of possible sub-types can be virtually unlimited and in Annotea the users could define types of annotations on the fly.

In AO we maintain the same mechanism, however we maintain a more conservative approach where a predefined set of annotation types are recommended. These are shown in Table 2. We also allow the implementation of types through a second mechanism called ‘composition’ which is based on multiple inheritance. We introduced it to foster reuse of the already available ontologies including SWAN [52,63]; it does not imply connecting classes but rather works by creating instances of both the *Annotation* class and the one we reuse, for instance, *swan:ResearchStatement* for a claim or hypothesis.

**Table 2:** AO Annotation types defined as sub-classes of the Annotea *Annotation* class.

Note	Note is a brief written record associated with a document or part of it. The body of a Note is usually free text and it can contain markup.
Errata	An Errata provides a correction of a document. The annotation body is the corrected version of the content or a description of the error.
Example	The annotation body represents the typical case of a class or group.
Definition	The annotation body contains a concise explanation of the meaning of a word, phrase, symbol or image to which the annotation points.

*Aot:Qualifier* is one of the key annotation types in AO and allows the definition of parallelism between AO and the Simple Knowledge Organization System (SKOS) model [32]. A *aot:Qualifier* (see Figure 4) defines a generic connection between the annotated online resource – HTML pages, digital images, audio files, etc. - or resource fragments and the URI of a term in an ontology or terminology. This mimics the relationship *skos:relatedMatch*. Qualifier SKOS-compatible subclasses that are: *aot:ExactQualifier* (Figure 4), *aot:CloseQualifier*, *aot:NarrowQualifier* and *aot:BroadQualifier*; these correspond respectively to the SKOS properties: *skos:exactMatch*, *skos:closeMatch*, *skos:broadMatch* and *skos:narrowMatch* (see Table 3).

**Table 3:** Additional annotation types can be used for creating SKOS like annotation.

Qualifier	skos:relatedMatch	Qualifier expresses the relationship between the object of the annotation and a well-defined representational unit. It allows a free text body. If only free text is present -this is equivalent to a tag.
ExactQualifier	skos:exactMatch	Used when the object of the relationship ao:hasTopic represents exactly the portion of the annotated document.
CloseQualifier	skos:closeMatch	Used when the object of the relationship ao:hasTopic represents closely but not exactly the portion of the annotated document.
BroadQualifier	skos:broadMatch	Used when the object of the relationship ao:hasTopic represents more broadly the portion of the annotated document.
NarrowQualifier	skos:narrowMatch	Used when the object of the relationship ao:hasTopic represents more specifically the portion of the annotated document.

**Figure 6:**
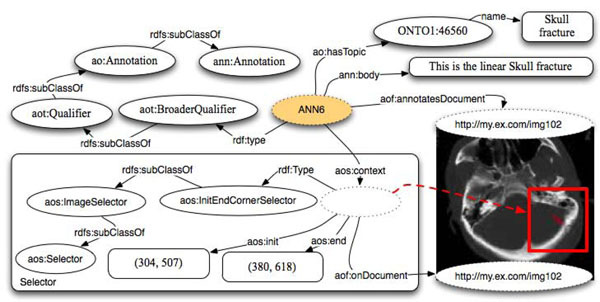
Annotation of a portion of an MRI image, using aot:BroadQualifier.

In Figure 6 we depict the annotation of a portion of an MRI image representing a linear skull fracture, using *aot:BroadQualifier*, a subclass of *aot:Qualifier*.

The annotator declared the image to express a ‘linear skull fracture’ through a textual label. As a textual label has a limited classification value and as the annotator could not find this specific term in the available ontologies/terminologies, she/he declared the image fragment to be represented by the term ‘skull fracture’ coming from a specific ontology and identified by a URI. As the annotator considers the term ‘skull fracture’ to have a broader meaning than what the image really expresses, the qualifier is declared to be broader than the ideal one. The parallelism with SKOS allows exploring automatic ontology building and improving the analysis of clouds of tags and annotations. In general, it is possible to either relate ‘a skull fracture’ or ‘the skull fracture of patient X’ to the portion of the image. The choice is left to the users and their specific needs. Also, when referring to raster images the accuracy of the selection is not as good as it can be with, for instance, with vectorial images. That is why the property *ao:hasTopic* might result having a different precision in different contexts.

In the same example it would be trivial to add another Qualifier defining as context the very same Selector. For instance we could state that the portion of the images also exactly represents an instance of the entity ‘Hematoma’; in this case we can use an *aot:ExactQualifier* in a similar way to that depicted in Figure 4.

In summary, we state that the area identified by the selector has a more precise meaning than the term “Skull Fracture” and has the exact meaning of the term “Hematoma” where both of these terms are entities specified in some other ontology/vocabulary. For images and fragments of images, it is not as easy to see the advantage of applying the SKOS approach as it is for text. For instance, if we focus on the example presented in Figure 4, where a chunk of text “BACE1” has been classified exactly as “Beta Secretase 1” from the PRO ontology, we can easily add, reusing the same selector, that the same chunk of text is classified as a narrower concept than a Protein in BIRNLex – the Biomedical Informatics Research Network (BIRN) project lexicon – which is identified by the URI: http://bioontology.org/projects/ontologies/birnlex#birnlex_23. As result, we could derive that the entity “Beta Secretase 1” in the PRotein Ontology (PRO) has a narrower meaning than the entity “Protein” in the BIRNLex vocabulary. As more annotations are attached to documents, we can infer cross-ontology relationships.

### Integration with SWAN and other existing ontologies

As mentioned above, as in Annotea, AO allows the creation of additional sub-types of annotations by sub-classing the Annotation class. In addition, in AO it is possible to define annotation by composition or multiple inheritance. An example is the SWAN Ontology which can be integrated with AO by defining an instance of both *ao:Annotation* and *swan:Claim* as depicted in the following RDF.

This second mechanism works well for integrating existing entities of other ontologies. The SWAN Ontology is just an example; there are others that we are already considering integrating such as CiTO [65] and BIBO [66], [65]which would be used for annotating citations.

### Curation

**Figure 7:**
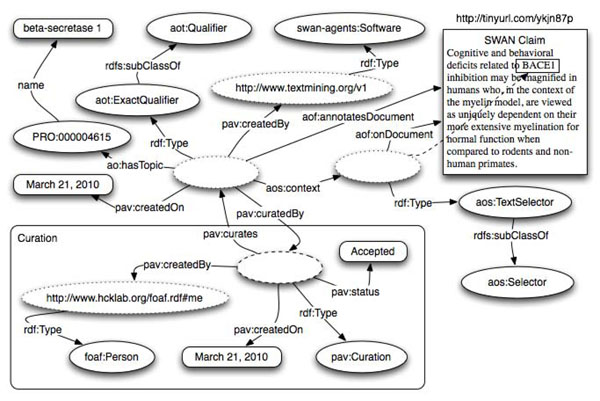
Curation token pav:Curation attached to automatically generated annotation denotes acceptance by the curator.

Curation is a crucial aspect of scientific publication and therefore an important aspect for our annotation ontology. In order to enable the complete cycle of activities that we define here as a RECS (Run, Encode, Curate, Share) process, AO supports a curation process that includes both manual annotation and text mining services. Figure 4 demonstrated an annotation generated by a text mining service, which is not yet evaluated and accepted by a human curator through what in AO is called ‘Curation Token’. In Figure 7 we display the same example with the curation token – some details such as those of the selector are omitted to keep the picture fairly simple. In the case depicted in Figure 7, a *foaf:Person* accepts the Software generated annotation.

**Figure 8:**
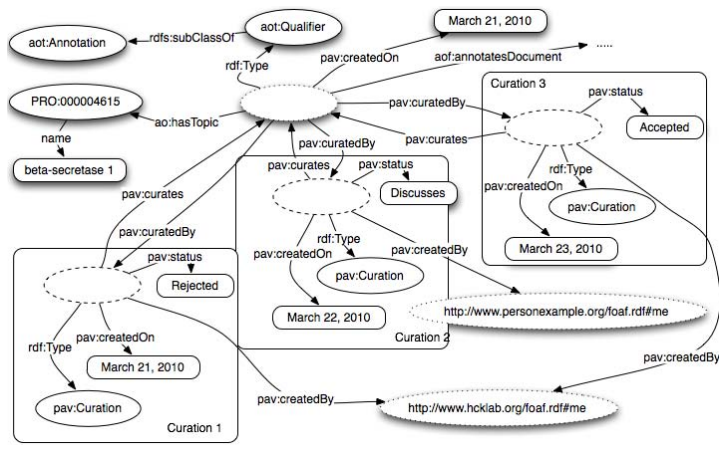
Multiple instances of the class pav:Curation on automatic annotation may be ordered via pav:previousCuration predicate.

In general, every annotation can undergo a multi-step curation process that can involve one or more users generating one or more curation tokens each. In Figure 8, we show a typical example of semi-automatic annotation workflow that can be summarized as follows: annotation is created by a text mining service, and first a user expresses a judgment on the validity of the automatic annotation, for instance "rejected" (curation #1). Later on a second curator might want to discuss the reason(s) for rejection (curation #2 with status: discussed). And finally a decision is taken and the annotation is ether rejected or accepted (curation #3 with status: accepted). We assume that the curation process is a linear story where the timeline can be determined through the curation dates. Alternatively, it is possible to compile explicitly an ordered list of curation item using for instance the SWAN Ontology module for collections [67].

### Annotation sets

In our experience, it is often useful to be able to group annotations by a specific criterion. Examples of criteria can be: the collection of all the annotation items related to proteins, the collection of all the annotation items representing scientific discourse elements, the collection of all the annotation items that have been published by a scientific community as officially curated. Also sets can be used to collect all the results by a specific text mining service, in this case the criterion would be the sharing of the same provenance. For grouping annotation items we introduced the concept of annotation set. The *ao:AnnotationSet* is a container of annotations.

### Versioning and evolution of the annotation

**Figure 9:**
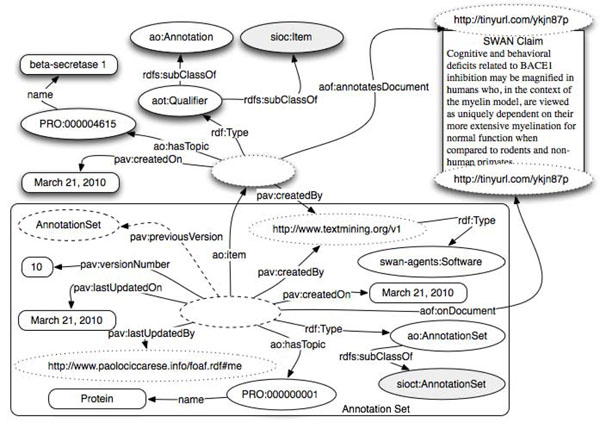
An ao:AnnotationSet containing a single annotation with mapping to SIOC.

When observing carefully Figure 9 it is possible to notice the presence of an object property pav:previousVersion and a datatype property pav:versionNumber. AO has been designed to be monotonic as much as possible. Therefore, once an annotation item is created in a set, it would be good practice to not be deleted by removal from the set. The term can be rejected through curation. Note that in Figure 9, the ao:AnnotationSet was created by a software agent and includes an automatically generated item.

Every new annotation item is created with a correspondent URI. Curation can be applied to the annotation. Edits of the annotation item, as well as new curation tokens, may (or may not) be defined as a new version of the annotation according to the requirements of the specific application. If a new version of the item is encoded, it will get a new URI and a pointer to the previous version of the same item. If multiple curation is performed a new item version will result having a longer curation chain as shown in Figure 8. An instance of an ao:AnnotationSet can be versioned every time the set of annotation items changes – a new item added - or even every time any item of the set changes. A new version of ao:AnnotationSet will result in a new item – with a new URI – pointing to the previous version of the same set.

It is also possible to derive one *ao:AnnotationSet* from another. This is common when a set that is publicly available is imported by an application and branched. In AO the second set will be connected to the first one through a relationship *pav:derivedFrom*. This will assure continuity to the annotation and the possibility to establish the correct attribution of the contributions. Branching one set into another is establishing the evolution of the annotation.

**Figure 10:**
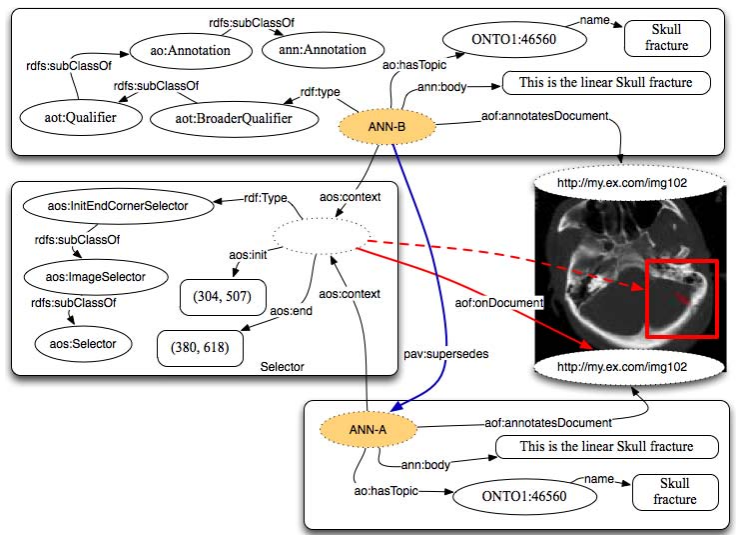
Evolution of an annotation, expressed using the relationship pav:supersedes.

Another example of annotation evolution happens when an annotation, initially defined as a tag, is later on attributed with a semantic entity (Figure 10 ). To keep the representation monotonic, we are going to define a second annotation with the *ao:hasTopic* property. The second annotation supersedes the first annotation that remains in the knowledge base.

### Alignment with the SIOC ontology

In Figure 8, besides the annotation set and its provenance and versioning, it is also possible to detect the mappings of the annotation ontology to the SIOC ontology [55,68]. SWAN and SIOC have been objects of an alignment process in the context of the Scientific Discourse Task Force (http://esw.w3.org/HCLSIG/SWANSIOC), one of the sub groups of the W3C Health Care and Life Sciences Working Group [69]. As creators of the SWAN ontology we confirm our commitment to keeping the two efforts aligned. The classes *ao:Annotation* and *ao:AnnotationSet* are declared sub-classes of respectively *sioc:Item* and *sioc:AnnotationSet*.

### Comparison to existing tagging ontologies

The scope of AO is much wider than tagging. However, tagging represents an important component for current online applications. We dedicated a fair amount of time trying to clarify the similarities/differences between AO and existing ontologies such as Newmann’s Tagging Ontology [44] and MOAT [56]

**Figure 11:**
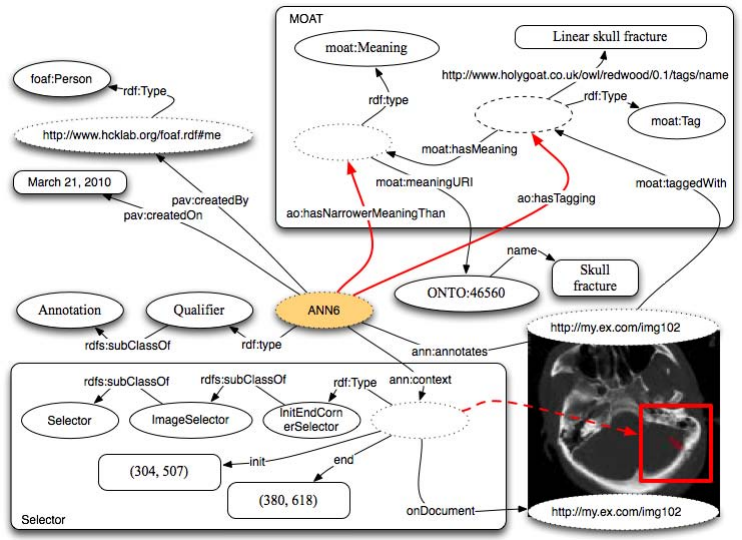
A free text tag attached to an MRI image with use of MOAT.

The MOAT (Meaning Of A Tag) project aims to solve the problem of ambiguous or redundant tags by providing a way for users to define meaning(s) of their tag(s) using URIs of Semantic Web resources. Even if AO was born to provide semantically defined data, we recognized the possibility of having a free text tag; we recommend this to be done natively leveraging the Annotea property ‘body’. Alternatively, it is possible to fully reuse the existing MOAT content in a way that increases its expressiveness and, at the same time, allows performing AO-style curation. The latter approach is depicted in Figure 11 where the free text tag “Linear skull fracture” is modeled using the *moat:Tag* class and attached to the annotation through the relationship *ao:hasTagging*. We haven’t reused the relationship *moat:taggedWith* because, as depicted in the same picture, it is supposed to link the annotated resource to the instance of the *moat:Tag* class.

It is also possible to notice that a meaning of the tag has been expressed through an instance of the class *moat:Meaning*. As the connected meaning is more general than the actual tag, the annotation instance refers to the meaning instance through the property *ao:hasNarrowerMeaningThan*. As you can see, from the perspective of the annotation we can define something more precise than *moat:hasMeaning*. AO allows us to define different levels of meaning through a set of properties that can be mapped directly into the Simple Knowledge Organization System (SKOS) model [32]: *ao:hasRelatedMeaning*, *ao:hasExactMeaning*, *ao:hasCloseMeaning*, *ao:hasNarrowerMeaningThan* and *ao:hasBroaderMeaningThan*. As was done for the Qualifier annotation types, these properties can mapped respectively to *skos:relatedMatch*, *skos:exactMatch*, *skos:closeMatch*, *skos:narrowMatch* and *skos:broadMatch*.

The model in Figure 11 can be translated or expressed – if the annotation does not derive from preexisting MOAT content - into pure AO annotation using qualifiers and the previously introduced SKOS-compatible subclasses: *ExactQualifier*, *CloseQualifier*, *NarrowQualifier* and *BroadQualifier*. This is the case of Figure 6 where this example is expressed in pure AO.

Besides the possibility of reusing and improving the existing MOAT content in AO, it is also worth mentioning how the annotation type Qualifier, that in RDF can look like:

can be transposed in MOAT and Newmann’s Tagging Ontology as a tag:RestrictedTagging, which represents the meaning of a tag in a specific context:

Once again, the scope of AO is much bigger than MOAT, but we wanted to provide integration with MOAT to be able to reuse already existing content. It is also possible to apply MOAT tags to any other annotation type - for instance a Note – even if we generally recommend creating multiple annotations pointing to the same document or selector. Doing so, it will be possible to perform curation for each specific piece of information.

## Conclusions

The AO model of web document annotation allows users, including journal or web community editorial staff, individual scientists, and computational web agents, to construct and persist scientific document annotation as RDF; making it possible to publish the annotation data as Linked Open Data as well as to query it by means of languages such as SPARQL, and to reason about it with SWRL. The AO ontology does not propose a new domain ontology nor tagging vocabulary to represent activities within folksonomies; on the contrary, it reuses and extends existing approaches such as Newman’s Tagging Ontology and MOAT, so that they can easily interoperate. It is also compatible with SCOT since it is built upon Newman’s ontology.

The AO ontology provides essential abilities for scientific web communities and publishers, including support for: (1) building position-aligned term enrichment – leveraging existing ontologies, biomedical ontologies in particular - into documents regardless of whether or not one controls the original content; (2) linking content across web communities and communities of scientific users, with shared metadata; (3) constructing searchable semantic metadata stores linked to documents in a standard way; (4) curation, with provenance, authoring and versioning of all annotations; and (5) human, algorithmic, and human-reviewed algorithmic annotation. In summary, AO allows bringing the Semantic Web to the masses under the form of annotations of several kinds – notes, definitions, qualifiers, … - including linking documents and document parts to terms in existing ontologies – particularly, but not necessarily only, scientific biomedical ontologies.

The AO model of scientific document annotation has been developed and tested in prototype and early-development versions of the SWAN Annotation Framework, an online application for both manual and machine-generated markup of web content developed in the context of the project SWAN 2.0 - Hypothesis Management for Drug Discovery – started in collaboration with a major pharmaceutical company. The same tool is currently under customization to be deployed for the NIF (Neuroscience Information Framework, http://www.neuinfo.org/) community.

## Competing interests

The authors declare that they have no competing interests.

## Authors' contributions

Paolo Ciccarese is the author of the Annotation Ontology (AO) and the main contributor to this paper. He is currently co-developing an Annotation Framework in tandem with AO that allows users to annotate online documents producing content in Annotation Ontology format.

Marco Ocana co-developed server-side and persistence software for the Annotation Framework and provided critical use cases and productive input to development of the Annotation Ontology.

Sudeshna Das is the project manager for the Science Collaboration Framework (sciencecollaboration.org) and contributed to the annotation use cases for a scientific community particularly the need to capture the provenance of semi-automatically generated annotations. She also contributed to the editing and revisions of the manuscript.

Leyla Jael Garcia Castro provided significant use cases for AO and wrote the section on folksonomic tagging. She also collaborated in the definition of the AO and MOAT integration.

Tim Clark conceived of and provided overall guidance for development of the Annotation Ontology and Annotation Framework projects, wrote the Introduction and Discussion sections of this paper, and had overall responsibility for editing it.
